# Introduction of “Teach and Work from Home” in Radiology during the COVID-19 Pandemic and Its Current Status: Implementation Research Done at Tikur Anbessa Specialized Hospital

**DOI:** 10.4314/ejhs.v33i2.22

**Published:** 2023-03

**Authors:** Tesfaye Kebede Legesse

**Affiliations:** 1 Radiologist and Body imaging subspecialist, Addis ababa University, Ethiopia

**Keywords:** COVID-19, teach and work from home

## Abstract

**Background:**

The current COVID-19 pandemic is forcing the world community to change how things should be done to avoid widespread transmission and containment of infection. Some countries are promoting “Work from Home” for disciplines like radiology” to prevent infection dissemination in hospital facilities. So the aim of this study is to introduce ‘work & teach and from home’ during the pandemic by establishing convenient virtual platforms for faculty members and students.

**Methods:**

This implementation research introduced a model “teach and work from home” during COVID pandemic. It was evaluated using a cross-sectional design to assess the effect of attending each session on the exam score of residents during the 1^st^ 2 months of the COVID-19 declaration in Ethiopia. Teaching and service activities that didn't require physical presence were identified and replaced with virtual activities. Additional faculty teaching activity was also introduced to compensate for the reduced radiology caseload.

**Results:**

Teach and work from home was introduced, and a total of 196 online teaching activities were conducted during the model's two-month introduction. Online attendance of teaching activities was shown to have a positive relation with exam scores for all levels of trainees.

**Conclusion:**

It was able to introduce teach and work from home model in the department of radiology at Addis Ababa university and it was shown that residents' retention of knowledge with the virtual platform was encouraging. It also brought a new experience in virtual teaching which is still practiced after the pandemic

## Introduction

Since the introduction of the COVID-19 pandemic, there was rapid dissemination of the pandemic until the wide implementation of safety measures and the recent introduction of COVID vaccine which has resulted marked decline in both transmission and mortality (1–3). Even if the exact transmission mode is not fully understood, recommendations were forwarded from areas where the pandemic is spreading, in addition to a few already published works on prevention strategies that worked in China (4, 5). One of the recommendations was social distancing, but the literature results also argue for special distancing and social closeness rather than distance. Restrictions on activities in Wuhan would probably help to delay the epidemic peak. Physical distancing measures were also shown to be effective (4). It was also revealed that school closure could substantially impact the spread of a newly emerging infectious disease transmitted via close (nonsexual) contacts (5). Staying at home to the greatest degree possible is also one of the recommendations forwarded by the CDC, WHO, and many countries (6, 7).

Few pieces of research are documenting the changes and measures taken regarding education, patient and staff safety, and research within the radiology department. These are mainly published from middle- and high-income countries (8–11). A study done in a US radiology teaching unit showed that technology integration for distance learning has promise in maintaining the teaching-learning process while applying social distancing. It was found to introduce new methods of learning in the future (12). The application of advanced technologies in a resource-limited setup like ours is one of the biggest challenges in bringing change to the existing educational and service activities.

## Methods

**Research setting**: Radiology residency teaching in the College of Health Sciences (CHS) at Addis Ababa University involves direct patient contact, which includes doing radiological procedures such as ultrasound, contrast radiograph procedures, and interventional radiology.

The program at our institution includes doing radiological procedures like the ultrasound (US), intervention, fluorography procedures, and interpretation of the findings, as well as interpreting other cross-sectional imaging like Computerized Tomography (CT) and Magnetic Resonance Imaging (MRI). About 200 radiographs (X-rays) are taken, and more than 120 patients are scanned with ultrasound in four rooms daily. More than 60 patients are scanned with CT and 20–30 in MRI. Weekly, about 20–25 US and/or CT-guided interventions are done in the department. All cases except ultrasound studies are discussed daily in groups in each departmental unit, constituting 10–12 people, including the consultant. So, one reporting room was used by 50–60 people at a time.

As part of the teaching-learning activities, we do have daily afternoon interactive teaching sessions from 1:30 — 3:00. On average, 2 cases are presented in the session. Others were morning interdepartmental sessions where challenging issues are discussed with the multidisciplinary team for management decisions and weekly resident seminars.

Most of the teaching and learning activities so far required lots of residents and staff to sit together to discuss cases and makes consultations.

During the early waves of COVID-19 pandemic, the radiology department canceled all interdepartmental teaching conferences, seminars, and daily afternoon imaging viewing conferences as a measure of infection prevention and control. Group teaching consultations, which are the main teaching method in the department, were also reduced to one-to-one consultations, which markedly impaired the teaching-learning process. In addition, most of the academic staff have group offices, and the limited spaces made the one-to-one consultations difficult to implement.

Furthermore, vulnerable staffs with comorbidities and risk factors were urged to stay at home. Alternative measures were required to involve such staffs to continue their activities from home.

Academic staffs were taking individual efforts and different means to maintain the residency teaching, but there was an urging need of redesigning our teaching activities in such a way that appropriate technologies were used to replace most of the activities which were unsafe for all patients, residents, and staffs.

This also applies to service provision where the most important challenge was reducing waiting time and patient crowding. Even if there is a relatively decreased number of patients came to the department as many non-emergency activities were canceled in the hospital, there was still so much crowding in the area due to the limited space in the department. In addition, the increased number of patients with suspected COVID-19 infection coming to the radiology imaging unit also required strict measures to maintain social distancing.

If alternative measures were not laid down, the department teaching and service would have been and g collapsed. So, this research was done with the objective of introducing the model ‘teach and work from home’ within the radiology department of AAU to sustain the teaching and service delivery. It also assessed the effect of online teaching on students' performance

**Design**: This study used implementation research that introduced teach and work from home model which was evaluated using a cross-sectional design to see the effect of absentee from intervention on exam score of trainees. Model was introduced after 3 months of Covid declaration, in Ethiopia [in June and July 2019]. Residents' attendance was taken, and the total attendance number was converted into a percentile. At the same time, the exam score was measured based on the exams given to the final exam of residents.

**Study setting**: The study was conducted in Tikur Anbessa Teaching Hospital, within the Radiology Department, school of Medicine, Addis Ababa University. The department has a three years postgraduate program and the years designated as R1/Year 1, RII/Year 2 & RIII/Year 3. There are a total of 25 academic staff conducting the teaching-learning process in the department

**Study population**: The source populations are all residents who were enrolled and on training during the study period and all academic staffs who were actively working during the time. The study populations were Year II & Year III residents who took the online teaching activity.

**Sampling**: There were a total of 76 residents in the department by the time this research was conducted. We took all year II and year III residents which are the primary presenters and discussants of the afternoon sessions and are candidates who were required to discuss morning case discussions. There were a total of 56 residents included in this study.

**Intervention**: The diagnostic study was focused on the following activities. The objectives of the existing department teaching-learning activities were reviewed to address those teaching activities and services that can be done remotely. All radiological services were also reviewed, and those activities that didn't need a radiologist's presence were identified. The radiology caseload was analyzed three months before and three months after the declaration of the first case of COVID-19 in Ethiopia.

The implementation Strategy consisted of the following activities. 1) The current radiology Picture Archiving and Communication software (PACS) was configured to support remote access. 2) The department's teaching and learning activities, which were conducted remotely, were programmed, and the number and duration of the remote teaching activities were documented regularly; 3) Mobile internet apparatuses that can be used for the implementation duration were given to 2/3^rd^ of the academic staff and resident representatives.

**Actions/measures taken**: The following measures were taken to achieve the stated objectives.

1) Acquire public IP and configure PACS for remote access: A public IP address is an IP address that can be accessed directly from the internet and is assigned by internet service providers, in our case, Ethiopian Telecommunication Corporation. A series of discussions were made with the college and university IT team to acquire the public IP. Finally, the value of acquiring Public IP was explained to the responsible body at the Ethiopian Telecommunication Corporation. Configuring PACS for remote access was accomplished after discussions were made with IT and MedWeb company engineers to get permissions. The MedWeb software was updated, and the company made troubleshooting. This enabled staff and residents to log into the hospital image server remotely. PACS workforce flexibly and ensured those self-isolating could still contribute to clinical work. Those in isolation can also work from home.

2) Accomplish remote teaching activities and service delivery: - Residents were divided into two groups to work on a schedule so that it is easier to isolate a specific group if there is a COVID-19 suspect based on the hospital guideline to reduce institutional crowd and reduction of infection transmission. The residents who are not around will attend sessions and consultations online. The faculty members were also arranged in groups to work alternatively.

The different videoconferencing software were assessed and initially, a virtual platform was created by the principal investigator using Zoom link and all residents and faculty members were invited to join the platform. After two weeks of implementation the virtual platform was changed to Google Meet. The major reason was that the university encouraged us to use google meet because their IT staff are familiar with the system, and it was easier for them to provide technical support for the university community. In addition, Google Meet supports a larger number of participants, which was limited to 100 participants in the free version of the Zoom video conferencing platform and unlike the free Zoom, Google Meet can go on for an unlimited time frame. Google Meet can also be integrated with Google Classroom, and all video conferencing can be recorded and stored. Online attendances can also be collected automatically and stored on google drive.

Initially, brief orientation was given to the residents and faculty members by IT professionals of the university and schedules of the online activities. Topics were communicated via telegram messenger. Later institutional email address was used the send conference link to all residents and faculties. This made the process easier as daily regular morning and afternoon sessions can be accessed only with one invitation at the start.

After establishing the means of videoconferencing, the daily morning and afternoon sessions were restarted. This was also followed by the commencement of the interdepartmental MDT meetings and daily consultations. Also online activities were recorded and stored in Google drive

3) Modifying afternoon teaching sessions: Two major reforms were made to improve afternoon sessions. The first was to increase the number of cases to be discussed from 1–2 cases to 4–6 cases at a time. The second measure was to avoid unnecessary scrolling of the large volume of DICOM data. Representative images from DICOM were selected and displayed on PowerPoint for everyone to see clearly and be focused on the major radiological findings and clues.

4) Introducing staff case review sessions: Staff case review sessions were introduced, which were not part of the teaching activity before the pandemic. The staff case review sessions were done four times a week in the morning from 8 am – 9 am, where faculty members discuss teaching cases from their case collections. The session is interactive like the afternoon teaching sessions. Cases to be addressed to the residents were decided by the presenter based on the type and complexity of the cases.

5) Support other institutions with radiology teaching units: - Residents in other radiology teaching institutions were given access to the daily online activities of the department. This was possible because most teaching activities were similar and on the same schedule.

**Data collection and analysis**: The videos recorded from online video conferencing were collected and analyzed for the number of presentations/week and the number and variety of cases using SPSS version 20. The total number of cases reported in a week was also assessed and categorized based on body parts. At the end of the second month, questions were prepared by selecting cases from the online presentations made within the two months after the start of the online teaching. Cases were categorized into year II & Year III residents based on the cases addressed for the respective years to discuss during the online case discussions. Attendance of the residents was taken in each session and was gauged on the percentage attending the sessions. Exams were administered for year II and year III residents simultaneously on the same day, and scores were analyzed using SPSS version 26. The results were displayed using line graphs. Pearson correlation coefficient was determined to see the association between online attendance and exam scores.

## Results

**Figure 1:**
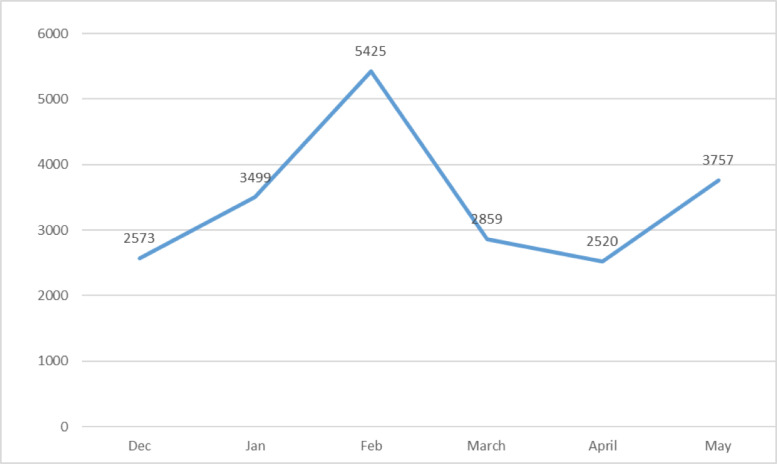
Total number of radiologic examinations done at TASH three months before and after COVID-19 in Ethiopia

**Radiology caseload**: There were 127829 studies saved in the department's picture archiving and communication software. Immediately after the declaration of the COVID-19 case in Ethiopia (March 1, 2020), there was a decrease in the radiology caseload, as seen in figure 1. Both the total number of cases and imagings done at specific modalities have decreased after COVID-19.

**Figure 2:**
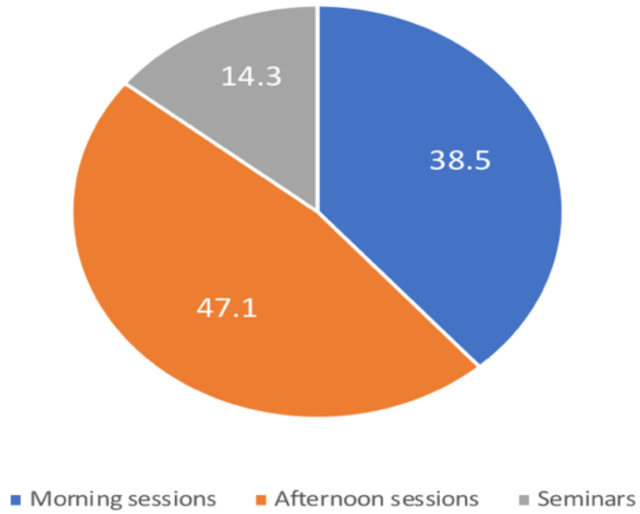
The proportions of time spent on morning and afternoon sessions and seminars.

**Teaching from home**: Sixty online sessions were made during the period of intervention, two months. Among these, 22 (36.7%) were morning sessions, 30 (50%) were afternoon, and 8 (13.3%) were seminars. The total duration of all online sessions was 48.8 hours. Among these, 18.8 hours (38.5%) were spent on morning sessions, 23hours (47.1%) spent on afternoon sessions, and 7 hours (14.3%) on seminars. Most of the online teaching sessions were conducted during afternoon sessions (Figure 2).

Among the eight online seminars, 4 were body imaging seminars, 2 MSK, one cardiothoracic imaging, and one neuroradiology seminar. A total of 196 cases were discussed during the study period, which is eight weeks. (Table 1). During both morning and afternoon cases, body imaging cases constitute the largest, and pediatric imaging cases include the smallest number of cases. 100 cases were discussed in the morning schedule and the rest 96 were in the afternoon schedule. On average there were 4.6cases and 4.4 cases discusses on the morning and afternoon schedule at a time.

**Table 1:** The number of teaching cases presented and discussed online in the morning and afternoon sessions per specialty units

Specialty units	Morning sessions	Afternoon sessions
Body	28	30
Neuro	22	26
Cardiothoracic	26	19
Musculoskeletal	15	14
pediatrics	9	7
Total	100	96

Most of the cases discussed in both the morning and afternoon sessions were CT studies and contrast radiographs were the least discussed modalities in both sessions (Table 2).

**Table 2:** The type of images presented and discussed online in the morning and afternoon sessions

Imaging	Morning session	Afternoon session
Plain X-rays	45	20
Ultrasound	7	6
Computerized Tomography	56	63
Magnetic resonance	20	21
Contrast radiography	6	2

**Figure 3:**
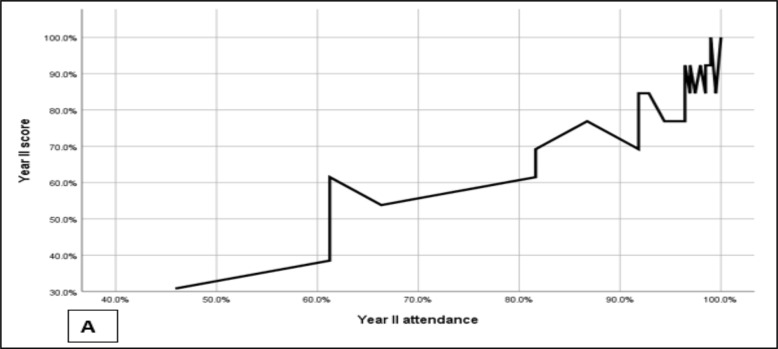
Exam scores of year II & year III residents after the introduction of teaching from home

**Figures 4:**
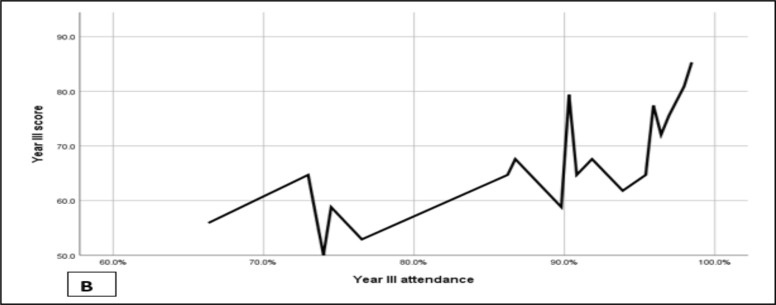
A & B: The correlation between attendance and exam scores for year II (5A) and year II (5B)

Even if home internet access apparatus was provided for 2/3^rd^ of the faculty members and three of resident representatives, there was repeated network interruption from the service provider during the study period exam scores and attendances. The mean exam score was 73.6% and the mean attendance (total of 196 sessions) was 89.9%. the Pearson correlation also showed a strong positive correlation between attendance and exam scores irrespective of the level of residency (Figures 3 & 4).

**Current status of teach and work from home**: Currently the department, where this model was introduced, has more than 16 teaching sessions every week and 25% of the activities are conducted using an online platform either alone or in combination with both face-to-face and online. This model enabled those who are away from the institution for different reasons to attend the teaching sessions virtually.

## Discussion

The pioneer of radiology training institution introduced teaching and working from home for radiologists and residents. All department teaching sessions, and service deliveries were effectively replaced with online activities. Immediately after the commencement of the online activities, it was possible to conduct a total of 48.8hours of online teaching activities within eight weeks.

Before the COVID-19 infection, radiology teaching in the institution where this research was conducted and in all other institutions found in Ethiopia was completely face-to-face. Except for a few incidents where partner institutions from other parts of the world were using online platforms to provide specific topics for our residents and a few attempts made decades back to start teleradiology service in the institution but local online education for radiology residency training was not practiced at all.

Even if most universities in developed nations, including china, changed the mode of learning from conventional to virtual learning even in the early phase of COVID-19 pandemic (13), developing countries like ours were too late to introduce online learning during the initial phase of the outbreak except closing schools for the time being

Immediately after the declaration of the first case of COVID-19 in Ethiopia, most schools, including undergraduate medical education, were closed. Still, medical residency training, including radiology, was continued, but there was a great challenge on how the teaching and service should continue without exposing the staff and residents. That is the turning point where the idea of teaching and working from home came into the picture.

Despite the practice of online radiology services and teaching were practiced in most developed institutions, introducing the model to our institution for the first time was not an easy task. The good part was that the network infrastructure in the college was well established and internet access from the institution was possible for all residents and staffs. As it was seen in most online teaching activities introduced during COVID-19 pandemic (14, 15), lack of home Internet access for faculty members and residents, poor internet access and repeated interruption of the service as well as lack of adequate online teaching experience were some of the challenges faced during the introduction. Home internet access for more than 2/3^rd^ of the staff and few residents were possible by providing mobile 4G internet apparatus through financial support granted from the University COVID-19 pandemic project fund.

With the introduction of online teaching, it was possible to completely replace the weekly imaging seminars and daily afternoon teaching sessions. The session was also modified to compensate for the reduced resident exposure due to the total reduction in caseload by increasing the number of cases to be discussed from 1–2 before COVID-19 to 3–5cases at a time. A total of 30-afternoon sessions were conducted, with 100 cases discussed from all specialty units. This improved the number and the variety of teaching cases to be discussed.

In addition to replacing all face-to-face teaching activities, staff case review sessions were introduced as one of the teaching models. A total of 22 staff case review sessions were made from home, and 100 cases were discussed during the 22 sessions with all specialty units were addressed. These created an opportunity for residents to look at the variety of cases that the faculty have collected for years and it partly compensated for the reduction of caseload.

Online education was found to have variable learner retention, and multiple factors affect retention during online education (16, 17). Despite all the challenges of our new teaching model and providing service from home, learner retention was good, as depicted in figure 4. One of the significant drawbacks of online education is the issue of attendance and inconsistency of a particular resident while attending an online education which is also the case in most other disciplines which used online platforms (18). At times it so happens that a particular resident was attending online education, but when the attendance is collected, they got disconnected due to network issues. Despite all these our result showed a positive correlation between attendance and retention of information. it was also shown in other studies that attendance correlated strongly with and had a significant effect on examination score(19). Even if COVID-19 pandemic negatively impacted education (20), it also made alternative means, like online learning, mandatory to maintain the teaching-learning process. This intern made us thought of introducing an online model or platform as part of the teaching learning method during the pandemic. The introduction of the model gave an opportunity to department faculties and residents to be familiar with online teaching systems evidenced by continuum use of the model for about 25% of the current weekly teaching sessions.

The COVID 19 pandemic, despite its downsides, came with a great opportunity for building resilience to adapt to change. It has opened the door for introducing newer and advanced ways of teaching and service in the radiology department. It opened the way for teaching and clinical practice by implementing PACS, which can be accessed from home. It was also an opportunity to function as a team with the different members of the staff. It also brought in new modes of learning which is still practiced after the pandemic. The author recommends further multicenter studies to evaluate factors that affect teaching and working from home to improve the continuum use of virtual platforms.

This is a single center study and poor internet connection during the implementation might also affect the outcome of this implementation
